# Pregnancy Complications and Impact on Kidney Allograft After Kidney Transplantation in IgA Nephropathy

**DOI:** 10.3389/ti.2023.11220

**Published:** 2023-05-04

**Authors:** Rikako Oki, Kohei Unagami, Jun Kakogawa, Hiroko Beppu, Taro Banno, Takafumi Yagisawa, Taichi Kanzawa, Toshihito Hirai, Kazuya Omoto, Kumiko Kitajima, Hiroki Shirakawa, Junichi Hoshino, Toshio Takagi, Hideki Ishida

**Affiliations:** ^1^ Department of Organ Transplant Medicine, Tokyo Women’s Medical University, Shinjuku, Japan; ^2^ Department of Urology, Tokyo Women’s Medical University, Shinjuku, Japan; ^3^ Department of Nephrology, Tokyo Women’s Medical University, Shinjuku, Japan; ^4^ Department of Obstetrics and Gynecology, Tokyo Women’s Medical University, Shinjuku, Japan; ^5^ Department of Nephrology, Ohkubo Hospital, Tokyo, Japan; ^6^ Department of Urology, Ohkubo Hospital, Tokyo, Japan

**Keywords:** kidney transplantation, graft survival, pregnancy, IgA nephropathy, pregnancy complications

## Abstract

Pregnancy in kidney transplantation (KT) recipients has been challenging because of the high risk of maternal, fetal, and renal complications. Although patients with immunoglobulin A nephropathy (IgAN)-chronic kidney disease (CKD) are at a high risk for hypertension in pregnancy (HIP), the maternal risk in KT recipients with IgAN as the etiology remains unclear. We retrospectively reviewed the medical records of pregnant KT recipients who delivered at our hospital. The incidence of maternal and fetal complications and the impact on kidney allografts between the group with IgAN as the primary kidney disease and the group with other primary diseases were compared. The analysis included 73 pregnancies in 64 KT recipients. The IgAN group had a higher incidence of HIP than the non-IgAN group (69% vs. 40%, *p* = 0.02). IgAN as primary kidney disease and interval from transplantation to conception were associated with HIP (OR 3.33 [1.11–9.92], *p* = 0.03, OR 0.83 [0.72–0.96], *p* < 0.01, respectively). The 20-year graft survival or prevention of CKD stage 5 in group with IgAN was lower than that in the group with other primary disease (*p* < 0.01). KT recipients should be informed of the risk of HIP and possibility of long-term worsening of postpartum renal function.

## Graphical Abstract

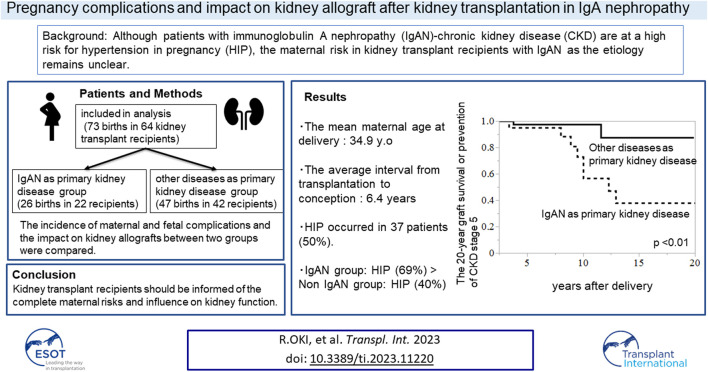

## Introduction

Female patients with end-stage kidney disease (ESKD) are known to have lower fertility rates due to disruption of hypothalamic-gonadal axis (1). Earlier studies have revealed that the probability of delivering a live-born baby may be rounded to 1:100 for women on dialysis compared to the overall Italian population; (2). Meanwhile, women with functioning kidney grafts have a 10-fold higher probability of delivering a live-born baby than patients on dialysis; (2). Thus, kidney transplantation (KT) deserves special attention because it provides a hope for women with ESKD who desire for childbearing. Along with increase in KT, increasing number of post-KT recipients in Japan who experienced pregnancy and childbirth have been observed, with over 500 cases (3, 4).

However, pregnancy in KT recipients remains challenging because it might severely affect graft kidney function, fetal development, and maternal health; in particular, the risk of deterioration of allograft function and/or occurrence of antibody-mediated rejection exists throughout pregnancy (5). Mohammadi et al. have reported that one-third had deterioration in graft dysfunction during pregnancy, more than 60% of which did not return to baseline (6). Pregnant KT recipients are also reportedly at a higher risk of gestational diabetes, hypertension during pregnancy (HIP), preeclampsia (PE), cesarean section, and preterm delivery (7).

Immunoglobulin A nephropathy (IgAN) is the most common glomerulonephritis, which is one of the leading causes of ESKD in the younger generation. Therefore, IgAN would also be the common primary disease of ESKD for KT recipients (8), in particular among young women of childbearing age.

In general, for pregnant women (non-KT patients), IgAN is considered as a risk factor for adverse outcomes. A systematic review by Piccoli et al. has revealed that the incidence of adverse pregnancy-related outcomes, including HIP or PE, was ten-fold higher in pregnancy with IgAN (non KT patients), than in control groups (9). In this study, baseline kidney function (estimated glomerular filtration rate [eGFR]) was relatively well preserved (9). Another study has reported that pregnant women with IgAN (non-KT patients) were at a higher risk of having preterm birth, PE and small for gestational age babies (10). However, whether IgAN might lead to an increased rate of adverse pregnancy-related outcomes in post-KT pregnancy remains unclear. There are a lot of KT female recipients with IgAN desire pregnancy. Therefore, clarifying the adverse pregnancy-related outcomes in KT recipients with IgAN may be necessary for preconception counseling in postpartum care.

This study was conducted to evaluate pregnancies in KT recipients and their impact on the mother, fetus, and graft function after delivery in our hospital. We focused on the difference in the incidence of maternal/fetal complications and the impact on kidney allograft, depending on the presence or absence of IgAN as primary kidney disease. We also examined the factors related to HIP in KT recipients.

## Materials and Methods

### Patients and Data Collection

This retrospective cohort study examined post-KT pregnant women who were on regular prenatal care and delivered at our hospital from 1 January 2001 to 31 December 2019. This study, conducted at a single center (Tokyo Women’s Medical University Hospital), was approved by the Institutional Review Board of the Tokyo Women’s Medical University Hospital (#2022-0084). This study retrospectively collected data from the medical records; therefore, informed consent was waived by the Institutional Review Board of the Research Ethics Committee of the Faculty of Medicine of Tokyo Women’s Medical University Hospital. All methods of research procedures were performed in accordance with the Declaration of Helsinki. The exclusion criteria were as follows: 1) follow-up period from delivery of <3 years; 2) pregnancies resulting in birth at <22 weeks of gestation; 3) miscarriage; 4) abortion; and 5) stillbirth. Stillbirth was defined as the intrauterine death of a fetus at ≥22 weeks of gestation.

The basic information of patients, pregnancy, and neonates (age at transplantation or pregnancy, information on transplantation, cause of ESKD, donor type, type of immune-suppressive therapy during the perinatal period, data on pregnancy/delivery/neonates, and kidney function pre-pregnancy/at postpartum/at 1 year and up to 3 years postpartum) were collected from the patients’ medical records. Latent IgA deposition from donor was not considered IgAN as primary disease.

Calcineurin inhibitors (tacrolimus or cyclosporine), mycophenolate mofetil (MMF) and prednisolone were used as maintenance immunosuppressants at our hospital. MMF was discontinued at least 6 weeks prior to the planned pregnancy and replaced with azathioprine (AZA), considering a risk of the teratogenicity. The date of preparation for pregnancy was defined as the date when MMF was discontinued or replaced with AZA on medical charts.

Pregnancy data included the incidence of HIP, gestational diabetes (diagnosed according to the recommendations of the International Society for the Study of Hypertension in Pregnancy [ISSHP] (11), cesarean section, gestational age at delivery, and preterm birth (babies born alive before 37 weeks of pregnancy). Blood pressure was measured with a brachial sphygmomanometer at home.

Data on neonates included birth weight, incidence of low birth weight (defined by the World Health Organization [WHO] as weight at birth of <2500 g), APGAR-score (Appearance, Pulse, Grimace, Activity, and Respiration), and umbilical cord blood pH at delivery. The maternal indications for cesarean section include severe HIP, deterioration of kidney graft function, prolonged labor (defined by the WHO as active labor that lasts >12 h (12), and previous cesarean delivery. The fetal indications for cesarean section included fetal growth restriction, fetal malposition, and non-reassuring fetal status. The interval between KT and pregnancy was calculated separately for each pregnancy.

Kidney function, including serum creatinine, eGFR, and data on proteinuria measured qualitatively, was evaluated pre-pregnancy, postpartum, and at 1 year and up to 3 years postpartum. The pre-pregnancy serum creatinine level was defined as the latest result within 3 months before conception. Postpartum serum creatinine levels were measured the day after delivery. Graft loss after pregnancy was defined as returning to dialysis or undergoing second transplantation. The indications for graft biopsy after delivery included time-dependent protocol biopsy or episode biopsy.

The Luminex single antigen beads assay (One Lambda Inc., Canoga, Park, CA, United States) was used to detect *de novo* donor-specific antibody (DSA). The assay was also conducted according to the manufacturer’s instructions as previously described. Positive DSA was defined as a mean fluorescence intensity >1000.

### Pregnancy Outcomes

This study compared the incidence of HIP between patients with IgAN as the primary kidney disease and those with other primary diseases. HIP is defined as chronic (predating pregnancy or diagnosed before 20 weeks of pregnancy) or *de novo* (either PE or GH), according to the ISSHP guidelines.

### Renal Outcomes

The primary outcome was the composite of all-cause graft loss and chronic kidney disease (CKD) stage 5 (eGFR<15 mL/min/1.73 m^2^) within 20 years postpartum. The occurrence and date of the first observed outcome postpartum were also investigated. For recipients who underwent twice deliveries, the duration from the first delivery was included in the analysis. Patient survival was examined at graft loss, the date when CKD stage 5 was detected, or the date of the last follow-up.

### Statistical Analysis

All statistical analyses were conducted using software (JMP^®^, Version<16.0>; SAS Institute Inc., Cary, NC, 1989–2021). Continuous data were expressed as mean ± standard deviation or median (interquartile range). Student *t*-tests or Mann–Whitney U-tests were used to compare continuous variables. The chi-square test or Fisher’s exact test was used to compare the categorical variables. Univariate and multivariate logistic regression analyses were performed to examine significant factors associated with HIP. The Kaplan-Meier method and log-rank test were used to compare differences in graft survival or CKD stage 5 within 20 years postpartum between groups. A paired-samples t-test was used to compare kidney function at each point (pre-pregnancy, delivery, 1–3 years postpartum). Values for which *p* was less than 0.05 were inferred as significant.

## Results

### Characteristics of Study Participants

**FIGURE 1:**
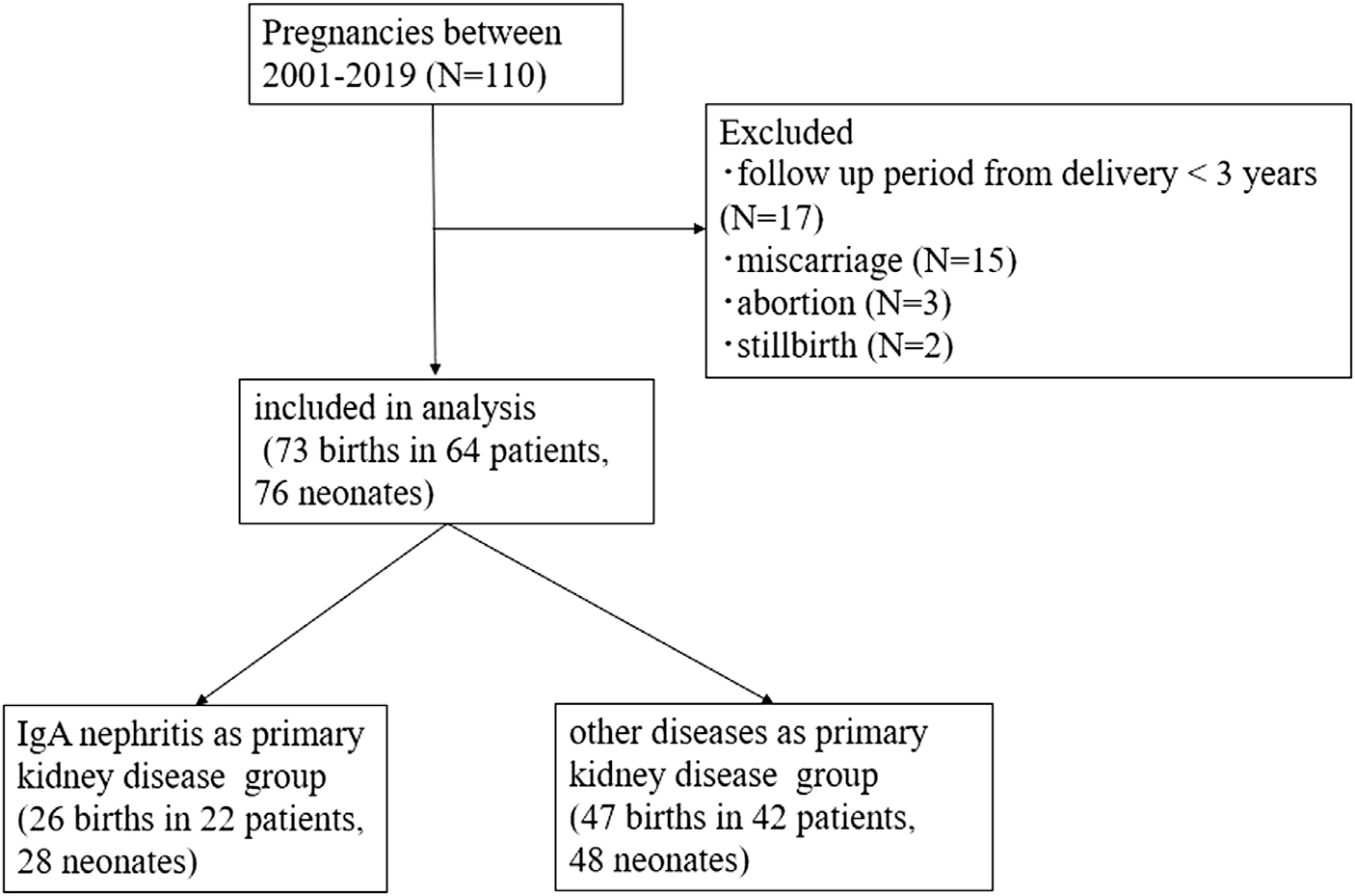
Chart showing flow of the study.

Of the 110 pregnancies during the study period, a total of 73 births in 64 patients were included in the analysis (Figure 1). Nine recipients experienced two deliveries, and three recipients delivered twins, resulting in 64 patients delivering 76 neonates by 73 pregnancies. In total, 26 births were recorded in 22 patients in a group with IgAN as the primary kidney disease, and 47 births were recorded in 42 patients in a group with other diseases as the primary kidney disease. The baseline characteristics of all participants are presented in Table 1. The mean patient age at transplantation was 28.1 ± 6.1 years old. The most common primary kidney disease was IgAN (*n* = 22, 34%), followed by glomerulonephritis (*n* = 13, 20%) [chronic glomerulonephritis, focal segmental glomerular sclerosis (FSGS), membranous proliferative glomerulonephritis, and rapid progressive glomerulonephritis], and congenital anomalies (*n* = 5, 8%). All the patients were recipients of living related KT, and most donors were the recipients’ parents.

**TABLE 1:** Basic information of the pregnant kidney transplant recipients (N = 64).

Variable	
Age at transplantation [y.o.]	28.1 ± 6.1
Primary kidney disease	
IgA nephropathy	22 (34%)
Glomerulonephritis^a^	13 (20%)
Congenital anomaly	5 (8%)
Vesicoureteral reflux	4 (6%)
Interstitial nephritis	3 (5%)
Diabetes mellitus	3 (5%)
Alport syndrome	2 (3%)
Others	3 (5%)
Unknown	9 (14%)
Living kidney donor	64 (100%)
Donor	
Parent	58 (91%)
Sibling	3 (5%)
Spouse	1 (1%)
Other	2 (3%)

^a^ Glomerulonephritis; except for IgA nephropathy. chronic glomerulonephritis (N = 5), focal segmental glomerular sclerosis (N = 4), membranoproliferative glomerulonephritis (N = 2), and rapid progressive glomerulonephritis (N = 1).

### Pregnancy Outcomes

Table 2 presents the comparison of pregnancy outcomes of IgAN between the primary kidney disease group and group with other primary diseases. Four patients exhibited IgA deposition in a zero-hour biopsy without mesangial proliferative changes. No significant difference in incidence of latent IgA deposition was observed between the two groups.

**TABLE 2:** Pregnancy outcomes stratified by groups according to whether IgA nephropathy was the primary disease.

Variables	All (*N* = 73)	IgA nephropathy (*N* = 26)	Non-IgA nephropathy (*N* = 47)	*p*
Latent IgA deposition in zero-hour biopsy	4 (5%)	3 (12%)	1 (2%)	0.09
Interval from transplantation to conception [years]	6.4 ± 4.0	6.2 ± 4.1	6.5 ± 4.0	0.77
<2 years	12 (17%)	4 (15%)	8 (17%)	0.86
2–5 years	17 (23%)	7 (27%)	10 (21%)	0.59
≥5 years	44 (60%)	15 (62%)	29 (62%)	0.74
Interval from preparation for pregnancy to conception [years]	1.1 ± 1.1	1.2 ± 1.1	1.0 ± 1.2	0.48
Average maternal age at delivery [y.o.]	34.9 ± 4.22	35.3 ± 3.39	34.7 ± 4.64	0.57
Immunosuppressive regimens during pregnancy				
TAC, AZA and PSL	52 (71%)	19 (73%)	33 (70%)	0.80
CyA, AZA, and PSL	2 (3%)	1 (4%)	1 (2%)	—
TAC and PSL	11 (15%)	1 (4%)	10 (21%)	0.046
TAC and AZA	3 (4%)	1 (4%)	2 (4%)	—
CyA and AZA	4 (5%)	3 (12%)	1 (2%)	—
CyA, MZ, and PSL	1 (1%)	1 (4%)	0 (0%)	—
hypertension in pregnancy	37 (50%)	18 (69%)	19 (40%)	0.02
Antihypertensive drugs during pregnancy				
Methyldopa	19 (26%)	10 (38%)	9 (19%)	0.62
Calcium blocker	1 (1%)	1 (4%)	0 (0%)	—
βblocker	1 (1%)	0 (0%)	1 (2%)	—
Antihypertensive drugs in follow-up period				
Calcium blocker	22 (30%)	11 (42%)	11 (23%)	0.84
Angiotensin receptor blocker	6 (8%)	5 (19%)	1 (2%)	0.06
Methyldopa	5 (11%)	2 (8%)	3 (6%)	0.68
Gestational diabetes	1 (1%)	0 (0%)	1 (2%)	—
Delivery				0.62
Vaginal birth	28 (38%)	9 (35%)	19 (40%)	
Caesarean section	45 (62%)	17 (65%)	28 (60%)	
Indication of caesarean section				0.27
Maternal	36 (80%)	15 (88%)	21 (75%)	
Fetal	9 (20%)	2 (12%)	7 (25%)	
Gestational age [weeks]	37.4 (32.7, 38.7)	35.0 (29.1, 38.3)	37.7 (34.1, 38.9)	0.04
Preterm birth	31 (42%)	14 (54%)	17 (36%)	0.14
Birth weight [g]^a^	2266 ± 783	2008 ± 887	2416 ± 681	0.03
Low birth weight^a^	42 (55%)	18 (64%)	24 (50%)	0.23
umbilical cord blood pH at delivery (livebirths)^a^	7.29 (7.25, 7.33)	7.28 (7.25, 7.31)	7.30 (7.26, 7.34)	0.14
APGAR score after 5 min^b^				0.03
7–10	67 (93%)	21 (84%)	46 (98%)	
4–6	3 (4%)	3 (12%)	0 (0%)	
0–3	2 (3%)	1 (4%)	1 (2%)	

AZA, azathioprine; CyA, cyclosporine; IgA, immunoglobulin A; MZ, mizoribine; TAC, tacrolimus; PSL, prednisolone.

Continuous data are presented as the mean ± SD or median (IQR).

Nine patients had two pregnancies. Each pregnancy was calculated separately.

^a^ Three patients had twins. Each neonate was evaluated separately.

^b^ Four values were missing for APGAR score in 5 min.

The average interval from transplantation to conception was 6.4 ± 4.0 years, and 60% of pregnancies were observed ≥5 years after transplantation. The average period from the date of preparation for pregnancy (discontinuation of MMF or replacement with AZA) to pregnancy was 1.1 ± 1.1 years. However, there were six cases, in which MMF was suspended or changed after pregnancy was confirmed. The mean maternal age at delivery was 34.9 ± 4.22 years old. No significant difference in mean maternal age was observed between the two groups. HIP occurred in 37 patients (50%), of which 14 patients had hypertension before pregnancy, and 23 patients developed *de novo* hypertension. As presented in Table 2, the most commonly used antihypertensive drug during pregnancy was methyldopa, which was subsequently replaced with a calcium blocker or angiotensin-receptor blocker.

The IgAN group had a higher incidence of HIP (69% vs. 40%, *p* = 0.02) and significantly lower gestational age and birth weight (mean gestational age; 35.0 weeks vs. 37.7 weeks, *p* = 0.04; average birth weight, 2008 g vs. 2416 g, *p* = 0.03) than the other primary diseases group. Additionally, more neonates in the IgAN group had a low APGAR score <7 at 5 min after birth.

Logistic regression models were used to evaluate factors related to HIP (Tables 3, 4). The analyses were adjusted for factors reported to be associated with an increased risk of HIP as follows (13, 14): maternal age at delivery, diabetes mellitus, CKD (kidney dysfunction before pregnancy), and interval from transplantation to conception. IgAN as the primary kidney disease and the interval from transplantation to conception were found to be related significantly to HIP in all the models (Table 4).

**TABLE 3:** Results of the univariate logistic regression analyses for hypertension in pregnancy.

Variables	OR (95% CI)	*p*
Age at transplantation	0.95 (0.85–1.06)	0.38
Primary kidney disease		
IgA nephropathy	3.32 (1.20–9.16)	0.02
Diabetes mellitus	0.47 (0.04–5.45)	0.55
Interval from transplantation to conception	0.85 (0.75–0.97)	<0.01
<2 years	3.54 (0.87–14.3)	0.08
2–5 years	2.12 (0.69–6.51)	0.19
≥5 years	0.28 (0.10–0.77)	0.01
Interval from preparation for pregnancy to conception	1.43 (0.89–2.29)	0.13
Average maternal age at delivery	0.95 (0.85–1.06)	0.38
Pre-pregnancy		
Cre	0.97 (0.13–7.30)	0.98
BUN	1.08 (0.98–1.20)	0.13
eGFR	0.99 (0.96–1.03)	0.77
Proteinuria	0.97 (0.13–7.31)	0.98

OR, odds ratio; CI, confidence interval; IgA, immunoglobulin A; Cre, creatinine; BUN, blood urea nitrogen; eGFR, estimated glomerular filtration rate.

**TABLE 4:** Results of the multivariate logistic regression analyses for hypertension in pregnancy.

Variables	Model 1		Mode1 2		Model 3	
	OR (95% CI)	*p*	OR (95% CI)	*p*	OR (95% CI)	*p*
IgA nephropathy	3.33 (1.11–9.92)	0.03	3.90 (1.29–11.8)	0.02	3.91 (1.28–11.9)	0.02
Interval from transplantation to conception	0.83 (0.72–0.96)	<0.01	0.84 (0.73–0.96)	<0.01	0.83 (0.73–0.96)	<0.01
Diabetes mellitus	0.29 (0.02–3.79)	0.35				
Average maternal age at delivery			0.92 (0.81–1.04)	0.16		
Pre-pregnancy Cre					0.48 (0.05–4.81)	0.53

OR, odds ratio; CI, confidence interval; IgA, immunoglobulin A; Cre, creatinine.

Model 1: IgA nephropathy, interval from transplantation to conception, diabetes mellitus.

Model 2: IgA nephropathy, interval from transplantation to conception, average maternal age at delivery.

Model 3: IgA nephropathy, interval from transplantation to conception, pre-pregnancy creatinine.

### Renal Outcomes

The median serum creatinine [mg/dL] level at pregnancy was 1.05, and the median eGFR [mL/min/1.73 m^2^] was 49.6. Only four cases (6%) had proteinuria (quantitative test 1+) before pregnancy (Table 5). Although eGFR [mL/min/1.73 m^2^] at delivery was significantly lower than that at pre-pregnancy (42.7 vs. 49.6, *p* < 0.01), it recovered to baseline level within 1 year postpartum (Table 5). Once renal function was recovered, it gradually and significantly deteriorated after 2 years, compared with that before pregnancy. No significant difference in renal function at each point was observed between the groups with IgAN as the primary disease (Table 5).

**TABLE 5:** Renal outcomes compared by groups according to whether IgA nephropathy was the primary disease.

Variables	All (*N* = 73)	IgA nephropathy (*N* = 26)	Non-IgA nephropathy (*N* = 47)	*p*
Pre-pregnancy				
Cre [mg/dL]	1.05 (0.95, 1.22)	1.16 (0.98, 1.33)	1.03 (0.94, 1.22)	0.20
BUN [mg/dL]	16.1 (13.6, 21.0)	16.7 (13.8, 21.3)	15.6 (13.5, 20.7)	0.41
eGFR [mL/min/1.73 m^2^]	49.6 (41.6, 57.6)	46.0 (38.0, 58.0)	50.3 (42.4, 57.7)	0.29
Proteinuria+	4 (6%)	2 (8%)	2 (5%)	0.56
Delivery				
Cre [mg/dL]	1.20 (0.98, 1.52)	1.27 (1.03, 1.48)	1.16 (0.96, 1.60)	0.77
BUN [mg/dL]	17.4 (12.5, 22.1)	17.7 (12.0, 21.9)	17.0 (13.1, 23.6)	0.90
eGFR [mL/min/1.73 m^2^]	42.7 (32.8, 53.6)	40.5 (33.3, 53.3)	45.9 (30.9, 54.4)	0.75
Proteinuria	28 (38%)	11 (42%)	17 (36%)	0.53
Proteinuria +	16 (22%)	8 (31%)	8 (17%)	
Proteinuria 2+	9 (12%)	2 (7%)	7 (15%)	
Proteinuria 3+	3 (4%)	1 (4%)	2 (4%)	
1 year postpartum				
Cre [mg/dL]	1.07 (0.91, 1.41)	1.13 (0.90, 1.39)	1.03 (0.90, 1.42)	0.82
BUN [mg/dL]	17.8 (13.3, 22.7)	17.4 (12.8, 22.7)	18.1 (13.6, 23.0)	0.62
eGFR [mL/min/1.73 m^2^]	48 (36.2, 61.8)	44.3 (36.3, 65.7)	49.3 (35.7, 61.5)	0.74
proteinuria	11 (15%)	4 (16%)	7 (15%)	0.44
Proteinuria +	8 (11%)	2 (8%)	6 (13%)	
Proteinuria 2+	3 (4%)	2 (8%)	1 (2%)	
2 years postpartum				
Cre [mg/dL]	1.08 (0.91, 1.34)	1.15 (0.92, 1.34)	1.05 (0.90, 1.35)	0.61
BUN [mg/dL]	18.0 (14.3, 22.8)	19.0 (13.8, 22.8)	17.3 (14.4, 23.1)	0.86
eGFR [mL/min/1.73 m^2^]	46.9 (36.5, 58.4)	44.3 (36.5, 56.5)	48.6 (36.3, 59.1)	0.51
Proteinuria	10 (14%)	5 (19%)	5 (11%)	0.30
Proteinuria +	9 (13%)	4 (15%)	5 (11%)	
Proteinuria 2+	1 (1%)	1 (4%)	0 (0%)	
3 years postpartum				
Cre [mg/dL]	1.09 (0.90, 1.42)	1.18 (0.98, 1.47)	1.03 (0.89, 1.41)	0.19
BUN [mg/dL]	18.0 (14.8, 23.1)	19.5 (16.3, 24.2)	17.0 (14.4, 22.7)	0.34
eGFR [mL/min/1.73 m^2^]	46.0 (34.1, 57.0)	41.2 (32.9, 57.5)	50.5 (35.0, 57.0)	0.19
Proteinuria	10 (14%)	4 (15%)	6 (13%)	0.35
Proteinuria +	9 (12%)	3 (11%)	6 (13%)	
Proteinuria 3+	1 (1%)	1 (4%)	0 (0%)	
Graft loss or CKD stage5 within 20 years postpartum^a^	10 (15%)	8 (36%)	2 (5%)	<0.01

IgA, immunoglobulin A; Cre, creatinine; BUN, blood urea nitrogen; eGFR, glomerular filtration rate; CKD, chronic kidney disease.

^a^ Evaluation based on pregnant kidney transplant recipients (N = 64), only the first pregnancies were analyzed.

Screening for panel reactive antibody was performed in 36 of 64 patients, and 8% (*n* = 3) of them had *de novo* DSA postpartum. No significant difference in the rate of *de novo* DSA was detected between the two groups (Supplementary Table S1) Kidney biopsy was performed in 36 of 64 patients postpartum. Biopsy-proven rejection developed in nine cases (chronic active antibody mediated rejection; N = 6, antibody mediated rejection; N = 3). The average interval from delivery to rejection was 5.7 years. More patients in the IgAN group had biopsy proven rejection than those with other primary diseases (50% vs. 12.5%, *p* < 0.01) (Supplementary Table S2).

**FIGURE 2:**
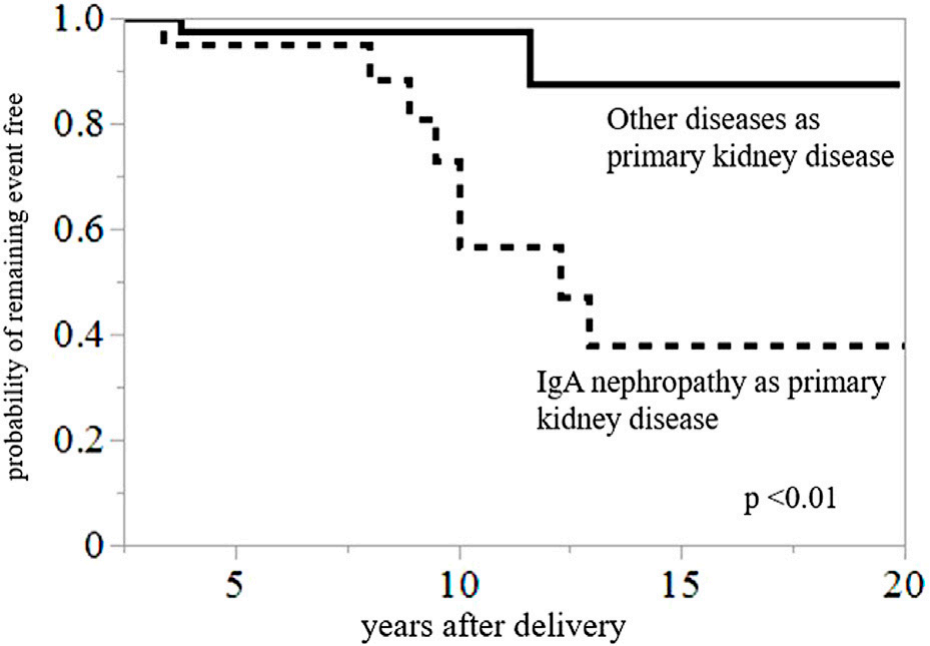
Kaplan–Meier analysis for composite outcomes of graft loss or CKD stage 5 within 20 years postpartum. Graft survival or probability of avoidance of CKD stage 5 during 20 years postpartum was significantly lower in the group with IgAN than in the group with other primary diseases (*p <* 0.01).

Among the patients in this study population, 10 recipients eventually experienced graft failure or progression to CKD stage 5 within 20 years postpartum. The causes of graft loss or CKD stage 5 included rejection (*n* = 2, 20%), recurrent IgAN (*n* = 1, 10%), calcineurin inhibitor toxicity (*n* = 1, 10%), and secondary (FSGS) (*n* = 1, 10%) and unknown causes (*n* = 5, 50%). Compared to the non-IgAN group, the IgAN group had a higher rate of graft loss or incidence of progression to CKD stage 5 [36% (8/26 patients) vs. 5% (2/37 patients), *p* < 0.01] (Table 5). Graft loss or progression to CKD stage 5 within 20 years postpartum was compared between the groups with IgAN as the primary kidney disease group and the group (N = 22) with other primary diseases (N = 42) using Kaplan–Meier analysis and log-rank testing (Figure 2). The results revealed that the 20-year graft survival or the rate of CKD stage 5 prevention in the IgAN group was significantly lower than that in the group with other primary diseases (*p <* 0.01).

## Discussion

In this retrospective observational study, we analyzed 73 pregnancies occurring in 64 KT recipients to examine factors related to HIP. The results of this study demonstrated that IgAN as the primary kidney disease and the interval from transplantation to conception were associated with HIP in KT recipients. Additionally, IgAN was significantly associated with a higher rate of graft loss or CKD stage 5 within 20 years postpartum, although no significant difference in the short term renal prognosis was noted. All patients were properly managed for kidney function and lifestyle-related diseases, including hypertension by attending physician during the perinatal and postpartum periods. To the best of our knowledge, this is the first study to demonstrate the possible contribution of IgAN to adverse pregnancy and renal outcomes in KT recipients.

The number of pregnancies among KT recipients reported worldwide has steadily increased, which has received considerable attention (15). Pregnant recipients are widely known to be at an increased risk for adverse maternal complications. In a review by Shah et al. of 6712 pregnancies in 4174 KT recipients, the mean maternal age was 29.6 ± 2.4 years (7), whereas it was 34.9 ± 4.22 years in our study. The rate of pregnancy outcomes was reported in a review by Shah et al. as follows; PE (21.5%), GH (24.1%), cesarean section (62.6%) and preterm delivery (43.1%) (7), which were compatible with our data, despite the high rate of late child bearing in our facility.

The prevalence of PIH in generally reproductive-aged women is approximately 7%–10% (16). HIP remains one of the major complications of pregnancy, which might cause maternal and perinatal morbidity and mortality (17). HIP-associated short-term complications include intrauterine growth restriction, small for gestational age, low birth weight and preterm birth (17). Although no significant difference in the rate of preterm birth or low birth weight was observed in our study, the median gestational age and mean birth weight were significantly lower in the IgAN group, than in the group with other primary diseases. (35 weeks vs. 37.7 weeks, *p* = 0.04, 2008g vs. 2416g, *p* = 0.03, respectively) Thus, it is presumed that these findings might reflect the high incidence of HIP in the group with IgAN.

Reportedly, the risk of GH or PE was 10 or 11 times higher in women with IgAN despite relatively well preserved kidney function (9). According to another report, IgAN was associated with an increased risk of preterm birth and cesarean section (10). Our pregnancy outcomes are in line with previous studies that have demonstrated the possible link of IgAN in pregnancy to adverse pregnancy outcomes.

Although the underlying pathophysiology of HIP has not been elucidated to date, placental ischemia and imbalance of angiogenic factors seem to be responsible for it (16). First, uteroplacental perfusion is reduced because of abnormal cytotrophoblast invasion of spiral arterioles. Next, placental ischemia leads to widespread activation/dysfunction of the maternal vascular endothelium, which contributes to enhance endothelial dysfunction (16). In particular, the release of antiangiogenic factors, including soluble fms-like tyrosine-kinase 1 (sFlt-1) and endoglin induces maternal vascular endothelial dysfunction (18). sFlt and endoglin reportedly increase before the onset of PE and correlate with disease severity (18). Zhai et al have demonstrated that excess sFlt-1 levels in patients with IgAN correlated with proteinuria and hypertension and elevated sFlt-1 levels contributed to endothelial injury in IgAN (19, 20). Circulating sFlt-1 levels are increased in various degrees of kidney dysfunction including post-KT (21). Moreover, a clinical association has been identified between circulating sFlt-1 and endothelial dysfunction in patients even after KT (21). An excess sFlt-1 level might be observed in post-KT recipients with IgAN, which could induce HIP. However, we did not obtain data of sFlt1 in our study, and we cannot substantiate it.

As for the activity or severity of IgAN before pregnancy, no significant differences in creatinine level and rate of proteinuria before pregnancy were observed between the groups with and without IgAN. Moreover, whether recurrent IgAN occurred in the kidney allograft in all pregnancy cases with IgAN remains unclear. Since 2019, we have routinely performed pre-pregnancy kidney biopsies to detect whether pregnancy was possible. However, the biopsy results before 2018 were insufficient because they were not determined in our hospital. Only 3 of 8 cases with pre-pregnancy biopsy results in the IgAN group had recurrent IgAN. Altogether, no evident mechanism between IgAN in KT recipients and HIP can be inferred from the results of this study.

In addition to the presence of IgAN, the interval from transplantation to conception was associated with HIP in KT recipients in our study. In the logistic analysis, the odds ratio associated with each 1-year increase in interval from transplantation to conception was 0.84 (95% confidence interval 0.73–0.96, *p* < 0.01), independent of maternal age. The optimal timing of pregnancy after KT remains controversial. The American and European guidelines for advising transplant recipients suggest that conception could be considered as early as 1–2 years post-transplantation, under a stable general condition (22, 23). Deshpande et al. have reported that obstetric complications including PE and gestational diabetes, were the highest in the <2-year interval following KT, and delivery outcomes including cesarean section rate and preterm birth rate were also less favorable in this interval (14). However, waiting the timing of pregnancy after KT might increase the risk of late childbearing and miss a chance of pregnancy because the fertility window could be narrow. Therefore, preconception counseling and care including family planning are warranted for safe and successful pregnancies in KT recipients.

Regarding the impact on postpartum kidney function, our results indicate that serum creatinine levels significantly decreased at delivery and then recovered at 1 year postpartum. A slight significant increase in serum creatinine level in 2–3 years was observed after delivery (Δserum creatinine 0.04 mg/dL, pre-pregnancy to post 3 years postpartum). This trend is similar as that previously reported (24). Buren et al have reported a rise in serum creatinine within 2 years postpartum of 0.18 mg/dL, which did not decrease for up to 10 years postpartum (24).

The rate of graft loss or CKD stage 5 within 20 years postpartum was significantly higher in the IgAN group than in the other primary disease. What is the rationale behind this result? First, the rejection rate in the IgAN group was higher than that in the group with other primary diseases (50% vs. 12.5%, *p* = 0.01). However, we cannot insist that recipients with IgAN are prone to rejection postpartum, because only 36 of 64 patients underwent kidney biopsy postpartum. Second, on whether IgAN as the primary disease affects graft survival, several studies have revealed that comparatively short-term graft survival (within 10–12 years) for IgAN patients was similar to that of patients with other primary disease (25, 26), but long-term graft survival (after 12–15 years) became worse (25, 27). Graft loss could be attributed to recurrent IgAN on long-term follow-up in their studies (25, 27). In our study, only one patient with graft loss or CKD stage5 within 20 years of delivery had recurrent IgAN. Third, the impact of bipara or twins may affect graft function. However, no significant difference in the percentage of recipients who had two deliveries or twins was observed between the two groups. Furthermore, the Kaplan–Meier analyses showed no significant difference in the 20-year graft survival or prevention of CKD stage 5 between unipara and bipara (*p* = 0.66). Collectively, whether IgAN as a primary kidney disease has a higher likelihood of deteriorating kidney function or graft loss after delivery is difficult to be explained. Pregnant recipients with IgAN should be paid special attention for kidney function postpartum.

The present study has several limitations. First, we might not have investigated or collected sufficient data on the unknown factors affecting the relationship between IgAN and HIP. Second, as mentioned above, the severity or activity of IgAN before delivery was not sufficiently considered because of the lack of data on kidney biopsy. We only had data on urinary protein as a qualitative test because quantitative tests are not routinely performed for general follow-up in outpatient clinics. Moreover, the race-dependent difference in IgAN was not considered as this study was performed in a single Japanese medical facility, which enrolls only Asian patients. Third, the generalizability of the results remains unconfirmed because this retrospective study was conducted in a single institution. Hence, larger studies including other facilities or different races are needed in the future to test our findings.

Within these limits, our study presents several clinical implications. Our findings suggest that female recipients of childbearing age wishing to consider pregnancy should be informed of the complete maternal risks and influence on kidney function by an expert multidisciplinary team. The best outcomes could be likely achieved under careful pre-pregnancy evaluation, planning, and perinatal management. We hope that our findings may guide periconceptional counseling on clinical decision-making and quality of life in KT patients.

## Data Availability

Raw data were generated at Tokyo women’s medical university. Derived data supporting the findings of this study are available from the corresponding author (KU) on request.

## References

[1] Shah S Verma P. Overview of Pregnancy in Renal Transplant Patients. Int J Nephrol (2016) 2016:4539342. 10.1155/2016/4539342 28042483 PMC5155089

[2] Piccoli GB Cabiddu G Daidone G Guzzo G Maxia S Ciniglio I The Children of Dialysis: Live-Born Babies from On-Dialysis Mothers in Italy-an Epidemiological Perspective Comparing Dialysis, Kidney Transplantation and the Overall Population. Nephrol Dial Transpl (2014) 29:1578–86. 10.1093/ndt/gfu092 24759612

[3] Abe T Ichimaru N Okumi M Imamura R Isaka Y Takahara S Pregnancy after Renal Transplantation: a Single-center Experience. Int J Urol (2008) 15:587–92. 10.1111/j.1442-2042.2008.02055.x 18462355

[4] Toma H Tanabe K Tokumoto T Kobayashi C Yagisawa T. Pregnancy in Women Receiving Renal Dialysis or Transplantation in Japan: a Nationwide Survey. Nephrol Dial Transpl (1999) 14:1511–6. 10.1093/ndt/14.6.1511 10383016

[5] Klein CL Josephson MA. Post-transplant Pregnancy and Contraception. Clin J Am Soc Nephrol (2022) 17:114–20. 10.2215/CJN.14100820 33731339 PMC8763163

[6] Mohammadi FA Borg M Gulyani A McDonald SP Jesudason S. Pregnancy Outcomes and Impact of Pregnancy on Graft Function in Women after Kidney Transplantation. Clin Transpl (2017) 31. 10.1111/ctr.13089 28805261

[7] Shah S Venkatesan RL Gupta A Sanghavi MK Welge J Johansen R Pregnancy Outcomes in Women with Kidney Transplant: Metaanalysis and Systematic Review. BMC Nephrol (2019) 20:24. 10.1186/s12882-019-1213-5 30674290 PMC6345071

[8] Aikawa A. Current Status and Future Aspects of Kidney Transplantation in Japan. Ren Replace Ther (2018) 4:50. 10.1186/s41100-018-0186-3

[9] Piccoli GB Kooij IA Attini R Montersino B Fassio F Gerbino M A Systematic Review on Materno-Foetal Outcomes in Pregnant Women with IgA Nephropathy: A Case of "Late-Maternal" Preeclampsia? J Clin Med (2018) 7:212. 10.3390/jcm7080212 30103519 PMC6111833

[10] Jarrick S Lundberg S Stephansson O Symreng A Bottai M Höijer J Pregnancy Outcomes in Women with Immunoglobulin A Nephropathy: a Nationwide Population-Based Cohort Study. J Nephrol (2021) 34:1591–8. 10.1007/s40620-021-00979-2 33683676 PMC8494659

[11] Brown MA Magee LA Kenny LC Karumanchi SA McCarthy FP Saito S Hypertensive Disorders of Pregnancy: ISSHP Classification, Diagnosis, and Management Recommendations for International Practice. Hypertension (2018) 72:24–43. 10.1161/HYPERTENSIONAHA.117.10803 29899139

[12] World Health Organization. WHO Library Cataloguing-in-Publication Data. WHO recommendations for augmentation of labour. Switzerland: Department of Reproductive Health and Research, World Health Organization (2014). Available at: https://apps.who.int/iris/bitstream/handle/10665/112825/9789241507363_eng.pdf?sequence=1 .

[13] Karumanchi SA Maynard SE Stillman IE Epstein FH Sukhatme VP. Preeclampsia: a Renal Perspective. Kidney Int (2005) 67:2101–13. 10.1111/j.1523-1755.2005.00316.x 15882253

[14] Deshpande NA James NT Kucirka LM Boyarsky BJ Garonzik-Wang JM Montgomery RA Pregnancy Outcomes in Kidney Transplant Recipients: a Systematic Review and Meta-Analysis. Am J Transpl (2011) 11:2388–404. 10.1111/j.1600-6143.2011.03656.x 21794084

[15] McKay DB Josephson MA. Pregnancy in Recipients of Solid Organs-Eeffects on Mother and Child. N Engl J Med (2006) 354:1281–93. 10.1056/NEJMra050431 16554530

[16] Granger JP Alexander BT Bennett WA Khalil RA. Pathophysiology of Pregnancy-Induced Hypertension. Am J Hypertens (2001) 14:178S–85s. 10.1016/s0895-7061(01)02086-6 11411754

[17] Kintiraki E Papakatsika S Kotronis G Goulis DG Kotsis V. Pregnancy-Induced Hypertension. Hormones (Athens) (2015) 14:211–23. 10.14310/horm.2002.1582 26158653

[18] Blom K Odutayo A Bramham K Hladunewich MA. Pregnancy and Glomerular Disease: A Systematic Review of the Literature with Management Guidelines. Clin J Am Soc Nephrol (2017) 12:1862–72. 10.2215/CJN.00130117 28522651 PMC5672957

[19] Zhai YL Zhu L Shi SF Liu LJ Lv JC Zhang H. Elevated Soluble VEGF Receptor sFlt-1 Correlates with Endothelial Injury in IgA Nephropathy. PLoS One (2014) 9:e101779. 10.1371/journal.pone.0101779 25007257 PMC4090210

[20] Zhai Y Liu Y Qi Y Long X Gao J Yao X The Soluble VEGF Receptor sFlt-1 Contributes to Endothelial Dysfunction in IgA Nephropathy. PLoS One (2020) 15:e0234492. 10.1371/journal.pone.0234492 32790760 PMC7425938

[21] Wewers TM Schulz A Nolte I Pavenstädt H Brand M Di Marco GS. Circulating Soluble Fms-like Tyrosine Kinase in Renal Diseases Other Than Preeclampsia. J Am Soc Nephrol (2021) 32:1853–63. 10.1681/ASN.2020111579 34155060 PMC8455271

[22] McKay DB Josephson MA Armenti VT August P Coscia LA Davis CL Reproduction and Transplantation: Report on the AST Consensus Conference on Reproductive Issues and Transplantation. Am J Transpl (2005) 5:1592–9. 10.1111/j.1600-6143.2005.00969.x 15943616

[23] EBPG Expert Group on Renal Transplantation. European Best Practice Guidelines for Renal Transplantation. Section IV: Long-Term Management of the Transplant Recipient. IV.10. Pregnancy in Renal Transplant Recipients. Nephrol Dial Transpl (2002) 17:50–5. 12091650

[24] van Buren MC Schellekens A Groenhof TKJ van Reekum F van de Wetering J Paauw ND Long-term Graft Survival and Graft Function Following Pregnancy in Kidney Transplant Recipients: A Systematic Review and Meta-Analysis. Transplantation (2020) 104:1675–85. 10.1097/TP.0000000000003026 32732847 PMC7373482

[25] Choy BY Chan TM Lo SK Lo WK Lai KN. Renal Transplantation in Patients with Primary Immunoglobulin A Nephropathy. Nephrol Dial Transpl (2003) 18:2399–404. 10.1093/ndt/gfg373 14551373

[26] Ponticelli C Traversi L Feliciani A Cesana BM Banfi G Tarantino A. Kidney Transplantation in Patients with IgA Mesangial Glomerulonephritis. Kidney Int (2001) 60:1948–54. 10.1046/j.1523-1755.2001.00006.x 11703614

[27] Moroni G Longhi S Quaglini S Gallelli B Banfi G Montagnino G The Long-Term Outcome of Renal Transplantation of IgA Nephropathy and the Impact of Recurrence on Graft Survival. Nephrol Dial Transpl (2013) 28:1305–14. 10.1093/ndt/gfs472 23229925

