# Supplementary material for: Pregnancy Complications and Impact on Kidney Allograft After Kidney Transplantation in IgA Nephropathy
Source: Transpl Int. 2023 May 4;36:11220. doi: 10.3389/ti.2023.11220 (PMC10193387; doi:10.3389/ti.2023.11220)
Supplement: Supplementary file 1 [file DataSheet1.pdf]

Supplementary Table 1. Results of the PRA screening test were compared by group according to whether IgA nephropathy was the primary disease.

*Screening test for PRA was performed postpartum in 36 of the 64 patients.

Variables	All (*N*=36)	IgA nephropathy (*N*=11)	Non-IgA nephropathy (*N*=25)	*p*
Test for PRA postpartum*				
Anti-Class Ⅰ antibody	12 (33%)	3 (27%)	9 (36%)	.61
Anti-Class Ⅱ antibody	12 (33%)	4 (36%)	8 (32%)	.80
De novo DSA	3 (8%)	2 (18%)	1 (4%)	.16

PRA, panel reactive antibody; DSA, donor-specific antibody; CKD, chronic kidney disease

Supplementary table 2. Result of biopsy compared by group according to whether IgA nephropathy was the primary disease.

*Postpartum kidney biopsy was performed in 36 of 64 patients.

Variables	All (*N*=36)	IgA nephropathy (*N*=12)	Non-IgA nephropathy (*N*=24)	*p*
Biopsy proven rejection	9 (25%)	6 (50%)	3 (12.5%)	.01
Recurrent glomerulonephritis	7 (19%)	4 (33%)	3(12.5%)	.14
Secondary FSGS Progression	6 (17%)	0 (0%)	6 (25%)	.058
of IFTA	3 (8%)	0 (0%)	3 (12.5%)	.20
No abnormal findings	11 (31%)	2 (17%)	9 (37.5%)	.20

FSGS: focal segmental glomerular sclerosis, IFTA; interstitial fibrosis and tubular atrophy
